# The interplay of olfaction and vision in host plant selection by *Anthrenus verbasci*

**DOI:** 10.1038/s41598-025-22240-7

**Published:** 2025-10-27

**Authors:** Ferenc Deutsch, Sándor Kecskeméti

**Affiliations:** Department of Chemical Ecology, Plant Protection Institute, Centre for Agricultural Research, HUN-REN, Budapest, Hungary

**Keywords:** *Anthrenus verbasci*, *Aegopodium podagraria*, Electroantennography, GC-EAD, Volatile collection, Behaviour experiment, Olfaction, Vision, Ecology, Ecology, Zoology

## Abstract

*Anthrenus verbasci* is commonly associated with museums, where the larvae can destroy valuable collections and displays. In households they are a nuisance; carpets, fabrics, food-items, or any proteinaceous material can be damaged. Little is known about the host preference of adults, they are frequently seen on plants of the Apiaceae, yet we do not know why they are preferred. Contrast of white flowers attract these insects primarily, yet it is unknown if floral volatiles mediate host recognition. We observed a high abundance of *A. verbasci* adults on the umbels of *Aegopodium podagraria* and sought to determine whether this mass attraction is driven by floral volatiles. Gas chromatography coupled electroantennography, revealed 8 antennally active compounds from the headspace volatilome of *A. podagraria*. Y-tube assays confirmed that intact *A. podagraria* floral volatiles attracted *A. verbasci* adults significantly; however, this attraction was reduced when damaged umbels were presented. In Petri dish experiments, germacrene-D induced aversion, highlighting a possible repellency. Beetles seemed to prefer discs baited with *A. podagraria* headspace volatiles at first, yet choice shifted towards the visual stimulus of dried umbellets at the end of the experiments. We have concluded that olfaction plays an important role in host recognition of *A. verbasci.*

**Supplementary Information:**

The online version contains supplementary material available at 10.1038/s41598-025-22240-7.

## Introduction

*Anthrenus verbasci (Linnaeus 1767)* (Coleoptera, Dermestidae) is a cosmopolitan insect found worldwide, it is common in the palearctic, oriental, saharo-arabian and sino-japanese zoogeographical regions^1^. *Anthrenus* is the most speciose genus of Dermestidae; with approximately 250 species described worldwide^2^. In nature, adults can be found on the flowers of several plant species, where they opportunistically feed on pollen and nectar^3^. Generally speaking, the larvae feed on dried protein material, thus its diet range can be quite extensive: from household items such as clothing, fabrics, carpet, food items, etc.^4,5^, to preserved insect or other animal specimens displayed in collections^6^. In seven zoological museums in India, *A. coloratus* caused extreme damage to the collections of 379 stuffed animal specimens^7^. Among the *Anthrenus* species, *A. verbasci* is one of the most frequent, since larvae can penetrate storage boxes through the smallest gaps and crevices^8^. According to a pest faunistic survey of 10 museum collections in Berlin, *A. verbasci* was the third most common species, while out of 9 museums in Vienna, it was the fourth most abundant among the museum pests^9^. In addition to museum collections, musical instruments have also been affected. On a certain clarinet, the larvae caused so severe damage that the instrument was rendered unusable^10^. Human health issues were also associated with A. verbasci. A 23-year-old woman living in Germany suffered an allergic skin reaction from the larvae of the insect^11^. In its natural habitat, the larvae are associated with bird and insect nests^3^, spider webs^12^, even with bat guano in caves^13,14^. Interestingly, the predatory behavior was also documented; the larvae feed on the eggs of the moth species *Lymantria dispar* and *Orgyia detrita*^15,16^. The species can be significant in agriculture, as larvae damage stored cereals and spices, reducing marketability^17^. In early spring from May, the emerging adults opportunistically feed on the pollen and nectar of almost any available flower^3^, but the adults are most frequently seen on the florets of umbelliferae^18,19^. The overall knowledge of the chemical ecology of *A. verbasci* is scarce, mainly pheromone studies of *Anthrenus* spp. were in focus, while host plant interactions were ovelooked. The sex pheromone of *A. flavipes*, (*Z*)-3-decenoic acid was determined as the first in the genus *Anthrenus*^20^. The decyl-butyrate has proved to be the sex pheromone of *A. sarnicus,* besides decanol^21^. The sex pheromone (*Z*)-5- and (*E*)-5-undecenoic acid released by females of *A. verbasci*^22^ has now also been identified. Plant volatiles also proved to be attractive for *A. verbasci*. *P*-anisaldehyde, a component often found in flowers, had a luring effect on both sexes. In addition, *p*-methoxyphenylacetone and *p*-ethoxybenzaldehyde were also similarly effective^23^. *Anthrenus* spp. adults prefer *Apiaceae* species as a food source where they forage for nectar and pollen. The white florets contrasting to the background serves as a visual stimuli that helps the beetles find a suitable host^18^. Visual signals are particularly relevant to *A. verbasci*, Goulson et al. demonstrated that the darker florets of the central umbellets of *Daucus carota* attracted more adults, compared to inflorescence where central florets were removed. Furthermore, replacing the darker florets with dead *A. verbasci* adults resulted in even greater attraction, whereas placing larger insects on the flower diminished this effect. It was proposed that the contrast between the darker central florets and the white outer florets serves as a crucial visual signal: darker florets potentially mimic *A. verbasci* adults^18^. Yet studies revealed that compounds like *p*-anisaldehyde induce attraction, meaning that olfaction too could be relevant. Overall knowledge of the chemical ecology of *Anthrenus* spp. remains limited, and there is no information about how olfactory and visual cues may interact in shaping their behaviour.

In the spring of 2023 and 2024 large numbers of *A. verbasci* adults were detected on umbels of *Aegopodium podagraria, Linnaeus 1753*. Although the presence of *A. verbasci* has been documented on *A. podagraria* before^24^, the olfactory background as a possible answer to the high attraction to this plant species has not been experimented with. The aim of this study was to identify compounds found in the headspace of *A. podagraria* that elicit antennal responses. Understanding chemical perception is essential, as it provides a basis for the development of attractant or repellent strategies aimed at manipulating behavior^25^. To uncover the physiologically active volatiles, gas chromatography coupled electroantennography (GC-EAD/FID) was used. Y-tube olfactometer assays were conducted to observe if plant-emitted volatiles alone are sufficient in attracting *A. verbasci*. Furthermore, because damage to plants is associated with the release of additional compounds, we repeated the experiments using mechanically wounded umbels, as the additional volatiles released may alter the behavior of insects. [26]. Moreover, since host plant localization in Dermestidae is primarily associated with visual cues, we examined whether the addition of a floral visual stimulus modifies the behavioral response previously observed to odor cues in Petri dish experiments.

## Material and methods

### Insect collection and species identification

**Fig. 1:**
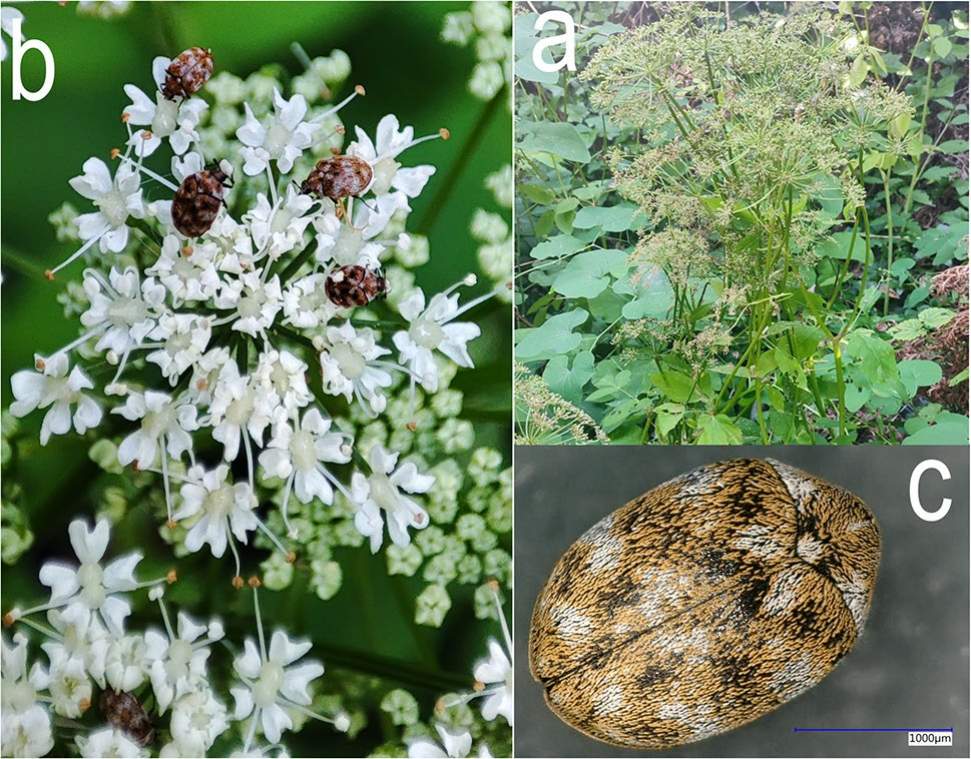
Showcasing pre-blooming *A. podagraria* (**a**) and blooming umbellets with foraging *A. verbasci* (**b**); focus-stacked image of *A. verbasci* (♂) (**c**)*.*

For electroantennography and behavioural experiments, *A. verbasci* adults were collected during May from a uniform patch of blooming *A. podagraria* plants located at N 47.54768°, E 18.93468°, Pest County, Hungary (Fig. 1a). The taxonomic identification of insect adults^3^ (Fig. 1b,c) and plant specimens^27^ were done by morphological identification. Voucher specimens of *A. verbasci* were collected and stored in ~ 70% EtOH, while *A. podagraria* specimen was placed in a herbarium. The voucher specimens are stored at Plant Protection Institute, HUN-REN ATK, Hungary.

### Volatile collection and mass spectrometry (GC–MS)

(1) Headspace volatiles were collected from the whole inflorescence of *A. podagraria*. Pre-blooming plants were dug up and potted individually in containers. Any dirt or external material was removed with water. Until blooming, plants were isolated with fine mesh nets to avoid unwanted insect colonisation. When blooming, the compound umbels (~ 10 umbels per plant) were covered with cooking bags (Hewa roasting bags, Germany). To avoid any damage to the plants, and to form an airtight seal, cotton pads mixed with dental wax were wrapped around the flower stem. The cooking bags were fastened to the cotton wrapped stem with zip-ties. Adsorbent filters were inserted inside the cooking bags from the top and connected with PTFE tubings (ID.: 5 mm) to a mobile open loop volatile collection system, which provided necessary airflow and air filtering (Volatile Assay Systems,USA). The adsorbent was 50 mg of HayeSep Q (60–80 mesh), and the airflow was set to 0.5 l min^−1^. The headspace was saturated for 30 min prior sampling and the total sampling time lasted for 1.5 h under laboratory conditions (24 ± 1 °C 60 ± 2% RH, two V-TAC G-series, 36 W, 4320 lm, 4500 K lightsource). Headspace sampling started at 10 am (GMT + 2) and was repeated three times on separate plants. From here forward, we will refer to the undamaged inflorescence of *A. podagraria* as ‘*intact umbels*’.

(2) Individual blooming umbellets were cut from the pedicel of *A. podagraria* and headspace volatiles were sampled immediately. We used plant materials described above. The sampled cut flower material was equivalent in quantity to ~ 10 umbels. Cut umbellets were placed in droplet shaped glass containers. The headspace was saturated for 30 min prior sampling. The glass container was equipped with ground joints, so a charcoal air filter (30 g) could be connected to it airtight, which supplied filtered air to the open loop system. The adsorbent used for volatile trapping was 50 mg of HayeSep Q (60–80 mesh) and was connected to the funnel end of the container with PTFE tubings (ID.: 5 mm). A constant air flow of 0.5 l min^-1^ was supplied by air pumps (Thomas G 12/02 EB, Garder Denver Thomas GmbH, Fürstenfeldbruck, Germany). The total time per sampling lasted for 1.5 h under laboratory conditions described above, and headspace was collected in three repetitions. We will refer to samples where we used cut umbellets as ‘*cut umbels*’.

Headspace sampling of isolated blooming *A. podagrari*a plants were only initiated, when we verified the presence of *A. verbasci* adults on *A. podagraria* flowers in natural conditions. The compounds captured by the adsorbents in both volatile collection method were eluted with 300 μl n-Hexane (purity 99.9%, VWR Chemicals) and stored at − 40 °C until electrophysiological experiments (GC-EAD/FID) and compound identification (GC–MS).

The methodology of chemical analyses was based on a previous study^28^. Plant volatiles were analyzed using gas chromatography coupled mass spectrometry (GC–MS) (HP Agilent 5890 GC and 5975 MS, Agilent Technologies). Samples were analysed on standard non-polar capillary columns, HP-5 UI (30 m × 0.25 mm × 0.25 μm, J&W). The injector temperature was set to 250 °C and operated in splitless mode for 0.5 min, the oven temperature was maintained at 50 °C for 1 min, then increased at 10 °C min^-1^ to 270 °C and held for 4 min. The flow rate of the helium was 1.0 ml min^-1^. Positive electron ionisation (EI +) was used, with an electron energy level of 70 eV, 2 scans s^-1^ were recorded in the range of 35–400 m/z. Compounds were tentatively identified by matching their mass spectra with those in the MS Libraries (NIST 23 and Wiley) using MassHunter (B.10.00, Agilent USA). Kováts retention indices were calculated for all compounds using C8-C20 alkanes calibration standards on HP-5 column. Calculated RIs were compared to RI values available in the NIST database. Furthermore, antennally active compounds’ fragmentation pattern, retention time and retention indices were also compared with those of synthetic standards previously used on the GC–MS system to verify identification. Identical volatile collection setups, comprising empty cooking bags and adsorbent filters, were additionally prepared as control systems. The volatile compounds detected in these control samples were subsequently subtracted from those identified in the plant headspace samples to ensure accuracy in the analysis.

### Electroantennography detection (GC-EAD)

To identify the electrophysiologically active compounds in the headspace of *A. podagraria* samples, gas chromatography coupled electroantennographic detections were carried out (GC-EAD/FID). The setup of instruments is identical as discussed in this study^28^. An Agilent 6890 N gas chromatograph (Agilent Technologies Inc., Santa Clara, CA, USA), equipped with an HP-5 capillary column (30 m × 0.32 mm × 0.25 μm, J&W Scientific, Folsom, CA, USA) and a flame ionization detector (FID) was used for separations. From the volatile samples, 2 μl was injected to the heated (220 °C) injector port in splitless mode. The starting oven temperature was initiated from 50 °C, held for 1 min, and constantly increased to 230 °C at a steady rate of 10 °C min^-1^. The Helium carrier gas was set to a constant flow rate of 2 ml min^-1^. The GC effluent was split equally in a low dead volume glass four-way splitter. Two pieces of deactivated fused silica capillary columns (100 cm × 0.32 mm × 0.25 μm) were connected to the four-way splitter; one led to the flame ionization detector (FID) (280 °C) and the other led to a heated (240 °C) EAD transfer line (Syntech, Kirchzarten, Germany) and into a glass tube (ID: 10 mm ) with a charcoal-filtered and humidified airflow of 1 l min^-1^ that was led over to the antennal preparation.

**Fig. 2:**
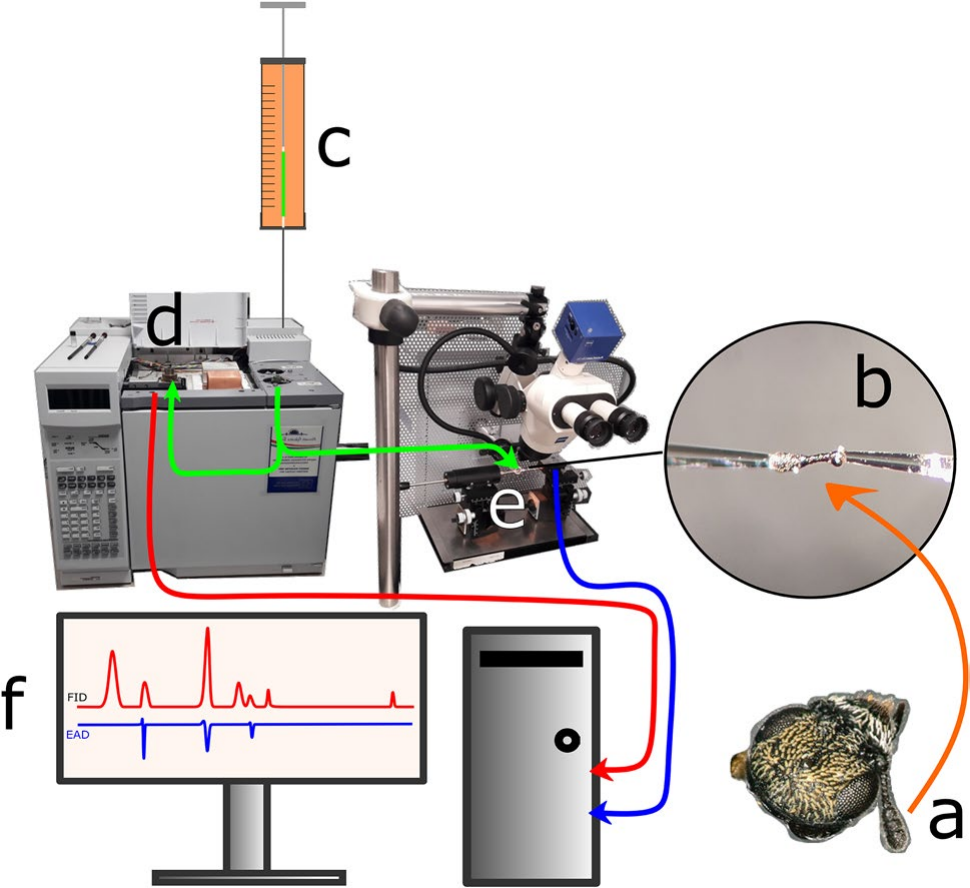
Schematic representation of gas chromatography-coupled electroantennographic detection (GC-FID/EAD). The antenna of *A.verbasci* (**a**) is excised and positioned between two glass capillaries (**b**) to establish an electrical connection. A volatile sample (**c**) is injected into the gas chromatograph, where individual compounds are separated. The effluent is split in a 1:1 ratio, directing one portion to a flame ionization detector (**d**), which generates an electric signal proportional to the quantity of ionized compounds (**f**). The other portion is delivered to the excised antenna via a humidified airstream (**e**). When a volatile compound interacts with its specific olfactory receptors, it elicits electrical potential changes in those receptors, and the summed activity across all responding receptors is subsequently amplified and recorded as an electroantennogram (EAG) signal (**f**).

During the electrophysiological readings, adults were prepared with the following method: The insect was held with soft grip-strength entomological tweezers with its ventral side facing upwards. This way the head was accessible, since *Anthrenus* sp. contract their body parts tightly when threatened. The head was slightly pierced in the middle with the apex of a no. 11 surgical scalpel-blade (Swann-Morton) and the caput was pulled from the thorax. From the head, one of the antennae was cut off with a surgical scalpel-blade (no. 11). For the recordings the removed antenna was held between two silica glass capillaries filled with Ringer’s solution^29^. The glass capillaries were fixed on silver electrodes that connected to a pre-amplifier (EAG Combi Probe, Ockenfels Syntech GmbH, Germany) where the recorded signals were converted in to digital (IDAC-2, Ockenfels Syntech) (Fig. 2). The recording software used was GcEAD (version 1.2.5 by Syntech), during recordings, external amplification was set to 9, and lower threshold (low cutoff) was set to 5 Hz. Experiments were conducted in five repetitions. Only responses consistent across all replicates were analysed further. The fundamentals of data preparation and analysis was based on Biasazin et al. 's work^30^. Purely on morphological keys, it is not possible to identify sexes of *A. verbasci* adults without dispatching them^3^. However the electrophysiological data did not indicate any discrepancies between individual’s volatile perception, therefore the data were pooled together.

### Behaviour experiments

#### I. Y-tube behaviour experiments

##### Experimental design

**Fig. 3:**
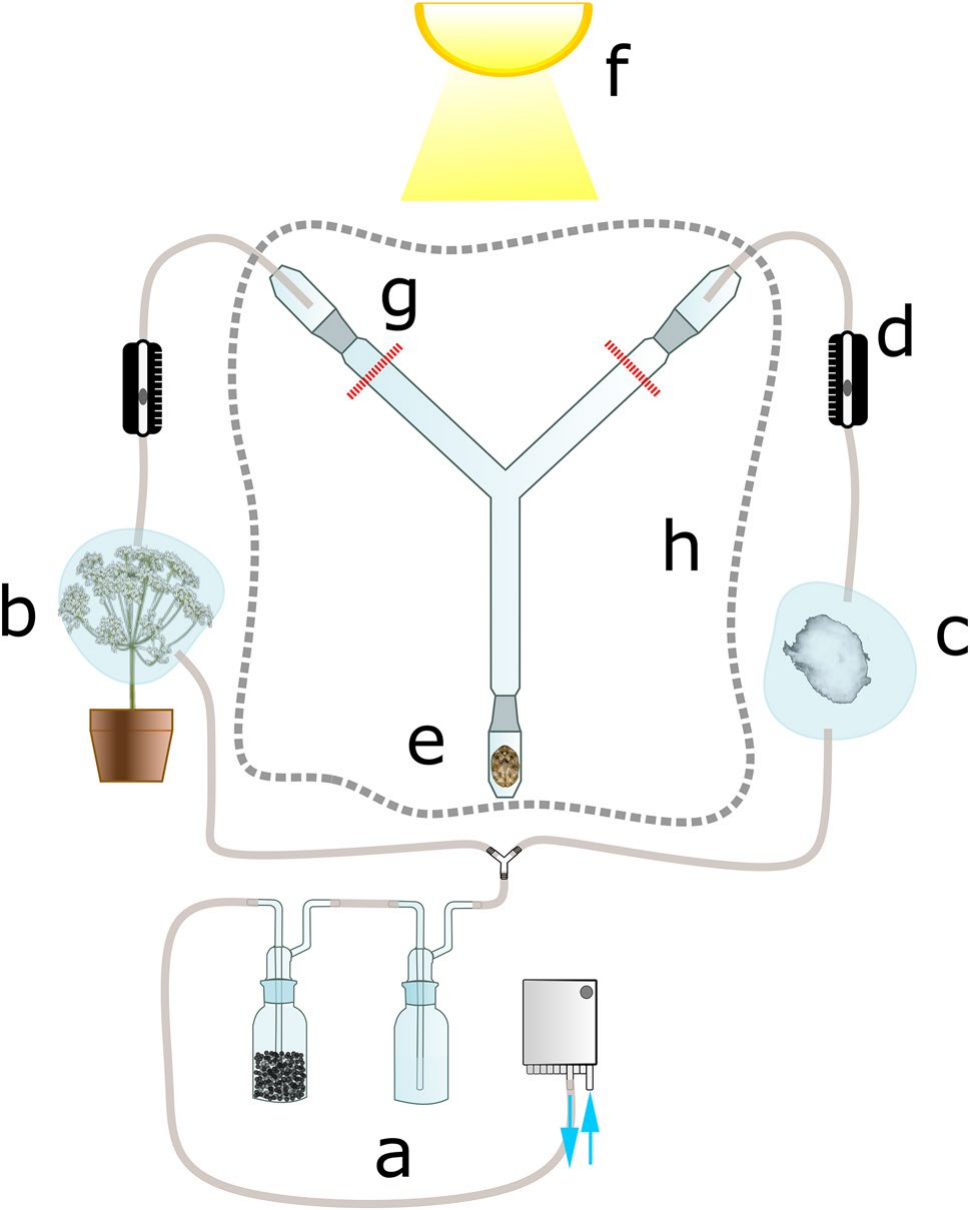
Schematic representation of the Y-tube olfactometer bioassay. Filtered and humidified air, supplied by an air pump (**a**), is divided into two streams directed toward the sample container (**b**) and the control container (**c**). Headspace volatiles from both chambers are conveyed through PTFE tubing into the olfactometer. Airflow balance between the two arms is maintained using adjustable flowmeters (**d**). The test insect is introduced at the starting position (**e**), where movement is stimulated by an LED light source (**f**). A choice is recorded if the insect traverses at least 80% of the total distance from the Y-tube bifurcation point (**g**) toward either arm. The Y-tube olfactometer was enclosed with a fleece barrier (**h**) to minimize external disturbances.

Bioassay experiments were performed in order to verify whether the attraction of *A. verbasci* towards *A. podagraria* is mediated by olfaction. Experiments were carried out in a Y-tube glass system. The constant airflow was supported via an air pump with PTFE inner coating. The effluent air was filtered with activated charcoal (50 g) and connected through a gas-bubbler filled with sterilized distilled water for humidification (80% RH). The filtered and humidified air was split into two lines, and each splitted line connected to a sample-container that held the test materials. The air flowing through the sampling-containers—carrying the volatilome—was connected to the end of the two choice arms (D: 16.5 mm, ID: 14 mm, common arm: 145 mm, choice arms: 125 mm, angle: 95°) with a threaded glass fitting. To ensure equal flow, adjustable flow meters were implemented (air-flow: 0.5 l min^-1^). Every tubing used in the setup was PTFE and glass components had Duran ground joints, ensuring airtight connection. Behind the Y-tube setup, an LED light source was placed (V-TAC G-series, 36 W, 4320 lm, 4500 K) approximately 1 m behind the experimental setup. Light stimulus was used during each experiment while no other luminous source was present. Experiments were carried out at 23 °C (± 1 °C) with 50–55% relative humidity (Fig. 3). In every experimental setup a single adult *A. verbasci* per repetition was placed at the end of the Y-tube and were given 5 min to choose. Prior to the experiments, insects were collected from nearby flora and were deprived from any food source for 48 h. Only responding specimens were taken into statistical analyses, but the number of non-respondents are presented in figures and referred to in the results section.The responsiveness of individuals was determined based on their movement in the Y-tube assay. An individual was considered responsive if it covered at least 80% of the distance from the bifurcation point, toward the volatile source. Since the sex of adults were not known, the Y-shaped glass where individuals traversed was washed with ≥ 99.9% acetone (Roth, Germany), after every measurement, and oven baked at 250 °C for 0,5 h after the 5th repetition of an experimental trial.

##### Experiments

I/(1) To ensure that there is no bias towards either arm in the Y-tube setup, a control experiment was carried out, where there were no volatile stimuli placed inside in either treatment holding container. A total of 20 insects were tested individually.

I/(2) *A. verbasci* adults were tested for their response to intact blooming umbel volatiles. Before the experiments took place, plants with ~ 10 pre-blooming umbels were placed in plastic pots individually and transferred to an experimental glass house. If any animals were present on the plants, then they were removed, the total plant surfaces was rinsed with 25 °C water, to eliminate dirt, possible animal excrement, honeydew residue, etc. Test plants were wrapped in fine plastic mesh as isolation for about a week, and monitored daily until full blooming. Before experiments, the florets of the plant were covered in a cooking bag. The floral stems were covered in a thick layer of dental wax and sterilized cotton-pads; the cooking bag was tightened with zip-ties (paying attention to not damage the plant) to ensure airtight sealing. PTFE tube from the air pump connected into the bottom side of the cooking bag, and a separate PTFE tube connected to the top side of the bag, which led to one of the arms of the Y-tube (with an adjustable flow meter in between). To the other arm an identical setup was connected, but instead of plant material, autoclaved and moist cotton balls were placed as control stimulus inside the cooking bag (Fig. 3). The two stimuli were not visible to the insects and were randomly assigned in every repetition. A total of 100 adults were tested in these experiments one by one.

I/(3) The third set of experiments were identical in design with the previously described setup, but instead of using intact umbels, freshly cut umbels were placed in the sample container used as olfactory stimuli. Every other parameter was the same as I/(2). In total 50 adults were tested in this experimental trial individually.

I/(4) Based on the GC-EAD/FID experiments, 8 compounds elicited consistent and strong antennal responses. The synthetic equivalent of these active volatiles were tested in behavioral experiments. The synthetic volatiles were mixed with each other in a 1:1 ratio and diluted in mineral oil to give a final concentration of 10 μg/μl per compound. During the experiments 10 μl of synthetic mixture was pipetted onto a filter paper (1 cm diameter) as chemical stimulus for *A. verbasci* adults. The filter paper was placed into a silica glass tube (15 cm long, ID.: 2 cm) with ground olive fittings placed at both ends. For control treatment, 10 μl of mineral oil was dispensed on filter paper and placed into an identical container. The synthetic compounds were (*α*-pinene ~ 70% mixture of isomers (PhytoLab); (-)-*β*-pinene ≥ 95.0%; ( +)-*β*-pinene ≥ 95.0%; *β*-myrcene ≥ 90%; *α*-phellandrene ≥ 75% (stabilised); (R)-( +)-limonene ≥ 99%; *β*-ocimene ≥ 90% mixture of isomers (Sigma-Aldrich/Merck); Germacrene-D ≥ 90.0% (MedChemExpress). In total the choice of 50 individual insects were recorded.

#### II. Petri dish experiments

##### Experimental setup

**Fig. 4:**
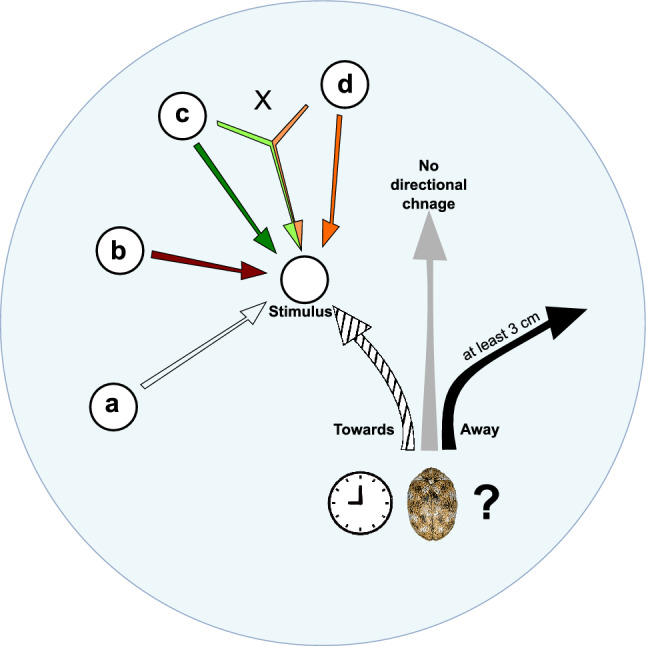
Concept of Petri dish behaviour experiments. Depending on the experimental trial, the response latency, movement direction, stay duration on stimulus, first–last choice, or travel distance of *A. verbasci* adults was recorded. Stimulus was filter paper which either contained nothing (serving as the control) (**a**), infused with germacrene-D (**b**), baited with the headspace volatile of *A. podagraria* inflorescence (**c**) or held dried *A. podagraria* umbellet on it (**d**). The “X” means that (**c**,**d**) treatment was introduced to the beetle simultaneously.

A silica glass Petri dish (ID: 150 mm) was used as an experimental arena where insects were placed. The collected insects were deprived of food for 48 h and kept at 23 ± 1 °C 60% RH and 16:8 L:D period. Only one beetle was placed in the arena per trial, and every experiment was repeated 20 times. Each trial involved placing a filter paper disc (D: 10 mm) inside the arena, treated with either a volatile sample, visual cue, both volatile and visual cues simultaneously, or left untreated to serve as a control (Fig. 4). A 10 min adaptation time was given to insects after placing them inside the arena, giving ample time to settle down. Each experiment lasted a maximum of 20 min, and every trial was recorded at a 1920 × 1080 resolution, 30 frames per second in .mp4 format with GoPro HERO10 camera (CHDHX-101) (GoPro Inc., San Mateo, California, USA). Experiments were conducted from 10 am (GMT + 2) at an average temperature of 24 ± 1 °C; air pressure around 766 ± 2 mmHg, with a relative humidity of 54 ± 2%. Two identical lightsources (V-TAC G-series, 36 W, 4320 lm, 4500 K) were placed directly above (3 m high) the experimental arenas and diffusers ensured that light was dispersed in the room equally. After every experiment the glass arenas were oven baked at 150 °C for 4 h, and beetles were discarded, using only naive specimens in further trials. The following incidences were recorded: Response latency—meaning the exact time when beetles changed its trajectory after introducing stimulus. Directional change—the possible direction a beetle could move relatively to the stimulus: away, towards or no directional change. Stay duration on stimulus—time spent by beetles on stimulus. First stimulus found and difference between final decision—Only recorded when odour and visual stimulus was compared simultaneously. Duration of 3 cm locomotion—the time needed to move 3 cm away in any direction compared to germacrene-D baited discs. A minimum of 1 min was held to ensure complete evaporation of hexane from the volatile samples before they were presented to the insects. The possible differences in experimental setups were as follows (Table 1).

**Table 1:** Experimental design and data of behavioural experiments.

Experiment code	Experiment design	Stimulus	Sample size	Data recorded
I/(1)	Y-tube	No stimulus (control)	20	Choice^(a)^
I/(2)		Intact umbels^(1)^	100	Choice^(a)^
I/(3)		Cut umbels^(2)^	50	Choice^(a)^
I/(4)		Synthetic blend	50	Choice^(a)^
II/(1)	Petri dish	Germacrene-D baited filter-paper	20	Response latency^(b)^ Directional change compared to stimulus^(a,c,f)^ Duration of 3 cm locomotion^(d)^
II/(2)		Odour stimulus^(3)^	20	Response latency^(b)^ Directional change compared to stimulus^(a,c,f)^ Stay duration on stimulus^(e)^
II/(3)		Visual stimulus^(4)^	20	Response latency^(b)^ Directional change compared to stimulus^(a,c,f)^ Stay duration on stimulus^(e)^
II/(4)		Odour and visual stimulus paired	20	Stay duration on stimulus^(e)^ First stimulus found and final decision^(c,f)^
II/(5)		Empty filter paper (control)	20	Response latency^(b)^ Directional change compared to stimulus^(a,c,f)^ Stay duration on stimulus^(e)^

In the footnote arabic numbers in superscript displays additional information about stimuli used in experiments, while letters in superscript refers to statistical analyses used in the experiments.

(1) 10 µl of A. podagraria headspace solution on filter paper.

(2) Dried umbellet of A. podagraria adhered to filter paper.

(3) Headspace volatiles eminating from undamaged full-blooming inflorescence of A. podagraria.

(4) Headspace volatiles eminating from full-blooming umbellets cut from the pedicel of A. podagraria flower.

(a) Chi-square goodness of fit tests.

(b) ANOVA (with Games–Howell post hoc test).

(c) Chi-square test of independence.

(d) Mann–Whitney U test.

(e) Kruskal–Wallis (with Dunn’s post Hoc test).

II/(1) To test the possible aversional effect of germacrene-D, we dispensed 10 µl of this compound (10 µg/µl dilution of n-Hexane > 99.9%) on a filter paper disc and placed it in the ~ 2 cm vicinity of the insect (but never directly in front of its path) and observed behavioural changes. We recorded the time needed for the beetle to move 3 cm away from the filter paper. For comparison, we placed 20 other specimens in empty arenas separately and timed the period when insects moved 3 cm without changing any direction serving as control movement duration data.

II/(2) To test the possible attraction effect of intact umbel headspace volatiles, 10 µl of intact *A. podagraria* flower volatile collection was pipetted on a filter paper and placed in the ~ 2 cm vicinity of the animal (but never directly in front of its path). The dispensed flower headspace onto filter paper is referenced as ‘odour stimulus’ from now on.

II/(3) The inflorescence of *A. podagraria* was separated to the individual umbellets and dried out at 40 °C for one week. This ensured that no plant odour remained and florets presented only visual stimuli (Figure S2). The absence of flower volatiles were verified by sampling the airspace of the prepared flower stimuli with solid-phase micro extraction (SPME) method for 1.5 h (DVB/PDMS/CAR coated fibers (StableFlex, 50/30 μm, Supelco, Sigma-Aldrich, Bellefonte, PA, USA)). The method of mass spectrometry used during SPME sample identification was the same discussed in this study^31^. These dried out umbellets were adhered with the mixture of cornstarch and water to the filter papers. This stimulus is referred to as ‘visual stimulus’ from now on. One dried out flower stimulus was placed inside the arena with one beetle. Like before, the visual stimulus was also placed in the ~ 2 cm vicinity of the insect (but never directly in front of its path).

II/(4) In this set of experiments two stimuli were present in the arena at the same time: One was the odour stimulus (II/(2)) and one was a visual stimulus (II/(3)) while one beetle was introduced in the arena. These two stimuli and beetle were positioned inside the arena so that the distances between them were equal, forming an equilateral triangle.

II/(5) In the control experiment we used the same filter paper discs as mentioned before, but without applying any volatile or visual treatment. Like before, the visual stimulus was also placed in the ~ 2 cm vicinity of the insect (but never directly in front of its path).

The full list of detailed behavioural experiments are shown in Table 1.

### Data analysis

#### Y-tube behavioural experiments

To see whether the distribution of frequencies of *A. verbasci* adults differs significantly we conducted multiple *Chi-square goodness of fit* tests with expected proportions of 1:1.

#### Petri dish behavioural experiments

In Petri dish experiments, to evaluate if there is a significant difference between the reaction latency of beetles across trials ANOVA was performed (α = 0.05). The raw data was transformed with log(x) function, and the normality of residuals were verified by *Shapiro–Wilk* test (*p* = 0.068). Since the homogeneity of variances failed, *Games-Howell* post hoc test was run to indicate differences in sub groups (α = 0.05).

To compare the time spent by beetles on stimuli (except germacrene-D baited papers) *Kruskal–Wallis* test was performed (α = 0.05) (since normality of residuals were not proven according to *Shapiro–Wilk* test *p* < 0,001), and differences in each subset was determined by *Dunn’s* post hoc test with *Bonferroni correction* (α = 0.05).

To statistically evaluate directional change of insects based on treatment effect (except simultaneous comparison of *A.podagraria* volatilome and visual stimulus of dried *A. podagraria* florets) *Chi-square* tests were performed. Comparisons of possible significance between trials were also done by *Chi-square* test of independence. Distribution within an experiment was analysed by *Chi-square goodness of fit* test expected frequencies of 1:1:1 or *One-Sample Binomial* test with hypothesised distribution of 1:1.

To highlight differences between the first choice of beetles during the comparison of odour and visual stimulus, *One-Sample Binomial* test was performed with hypothesised distribution of 1:1. To see how the final choice changed from the first one until the end of the experiment, *Chi-square* test of independence was done.

To compare the locomotion time needed to reach 3 cm in germacrene-D aversion experiment *Mann–Whitney U* test was performed.

Statistical analyses were performed using IBM SPSS Statistics ver. 22 (IBM Corporation, Armonk, New York, USA). In Y-tube experiments only actively responding adults were taken into statistical analyses. Figures and results also report the number of non-responding specimens however. In Petri dish experiments the data of perished individuals were discarded.

Heatmap visualization was created with R Studio ver. 2022.07.1 Build 554 (R Core Team, 2023) using the package “ggplot2”. Additional image editing was done using Adobe Illustrator (Adobe Systems, Mountain View, California, USA), InkScape vector graphics program 1.4 ((86a8ad7, 2024–10-11) and GNU Image Manipulation Program (GIMP 2.10.18). Transformation of raw data was done in Microsoft Office Excel 2016 (Microsoft Corporation, Redmond Washington, USA).

Video recordings were rendered with Camtasia software (TechSmith LLC., Michigan, USA) and behaviour of insects and incidents were analysed using Behavioral Observation Research Interactive Software 9.4.1^32^.

## Results

### Volatile collection and mass spectrometry (GC–MS)

We have identified 69 volatile compounds from the headspace of cut and intact *A. podagraria* umbels. We detected qualitative and relative areal differences between cut and intact umbels. The majority of identified compounds were terpenoids; from the 20 monoterpenoids the first five most abundant volatiles were: limonene (cut: 25.92%, intact: 17.81%); *β*-pinene (cut: 15.27%, intact: 16.28%); *β*-myrcene (cut: 8.93%, intact: 7.15%); *α*-pinene (cut: 8.27%, intact: 9.29%); (*Z*)-*β*-ocimene (cut: 2.65%, intact: 1.72%). We have identified 18 sesquiterpenoids, where the five most abundant compounds were: germacrene-D (cut: 14.94%, intact: 2.91%); *α*-farnesene (cut: 7.14%, intact: 1.35%); (*Z*)-*β*-farnesene (cut: 2.4%, intact: 0.78%); *α*-caryophyllene (cut: 0.63%, intact: 0.2%); *β*-elemene (cut: 0.49%, intact: 0.12%). We also identified 9 hydrocarbons, 8 aldehydes, 5 alcohols and 3 ketones. We have detected 12 peaks that were unique to cut umbel volatilome and were not detected in intact florets. The full list of detected peaks and identified compounds are listed in the supplementary material (Table S1).

### Electroantennography detection (GC-EAD)

**Fig. 5:**
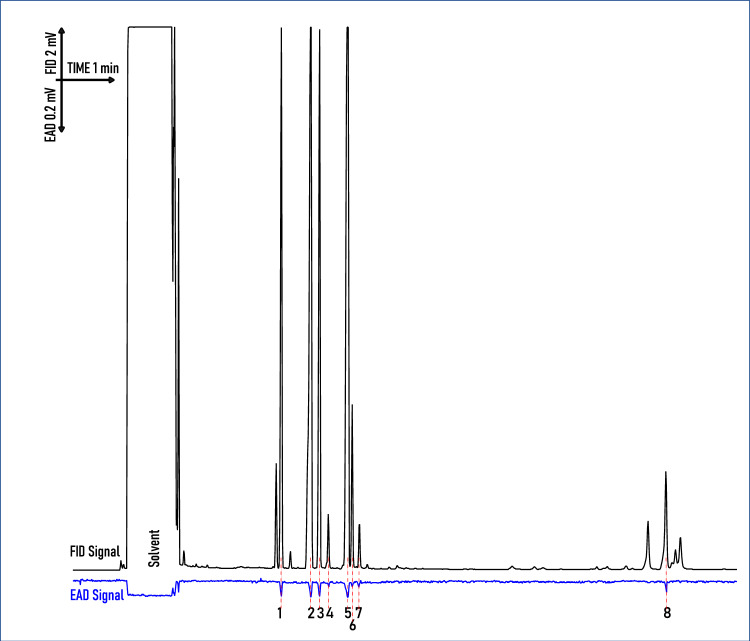
Representative Headspace volatiles compounds of *A. podagraria* umbels, detected by the flame ionisation detector (FID) (black trace) and EAD signal (blue trace) of potential changes in the antennae of *A. verbasc*i during gas chromatography coupled electroantennography (GC-EAD) recordings. Antennal responses are marked with arabic numerals (1–8) that align (dashed line) with a compound peak. The number of EAD peak was the same in the case of cut and intact umbel volatilome. (1: *α*-pinene; 2: *β*-pinene; 3: *β*-myrcene; 4: *α*-phellandrene; 5: limonene; 6: (*Z*)-*β*-ocimene; 7: (*E*)-*β*-ocimene; 8: germacrene D).

**Fig. 6:**
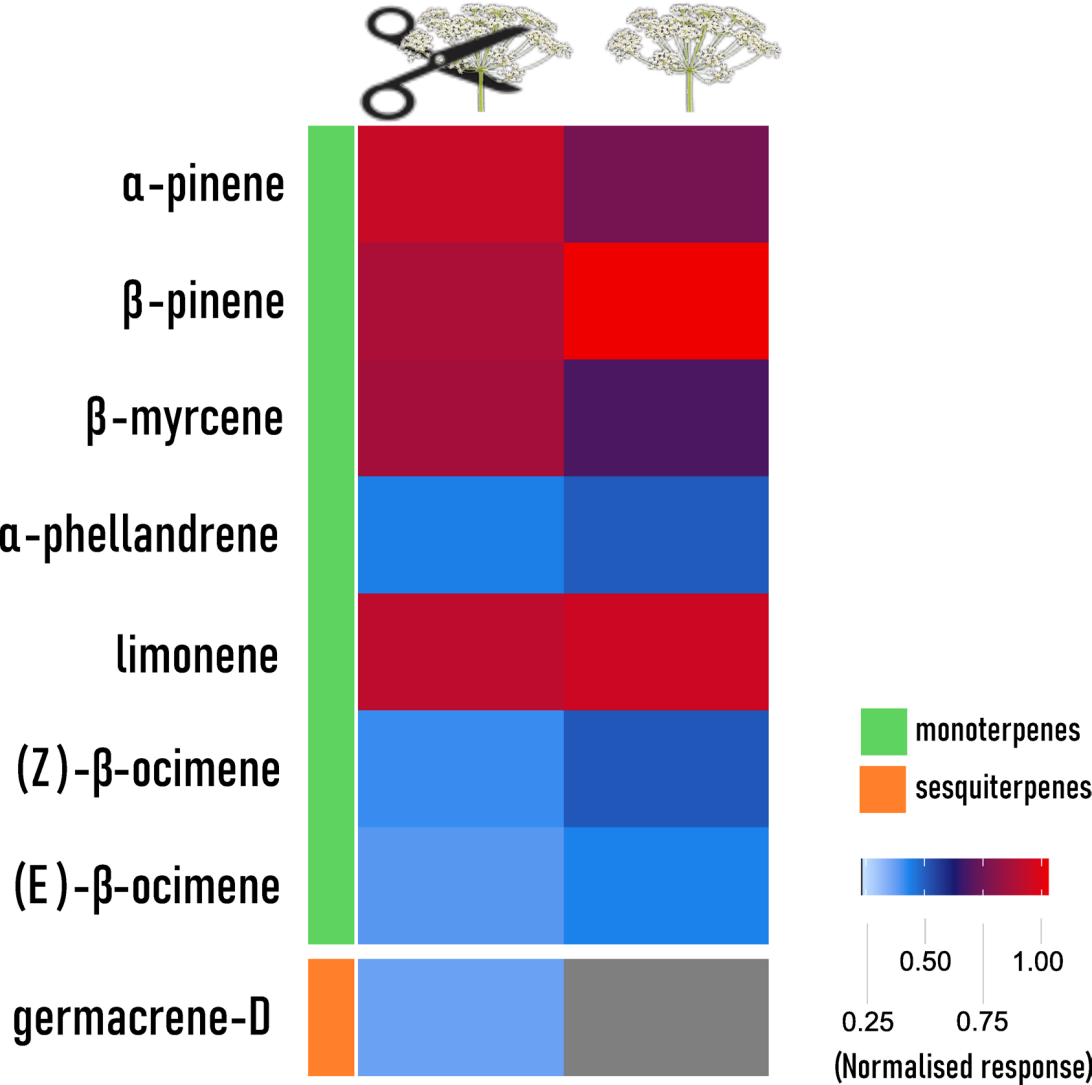
Heatmap illustrating antennal responses measured by GC-EAD using cut and intact headspace collection of *A. podagraria*. From left to right: columns show names of the identified volatile constituents, colour code for chemical classes, heatmaps of *A. verbasci* antennal responses to *A. podagraria* volatiles.

**Fig. 7:**
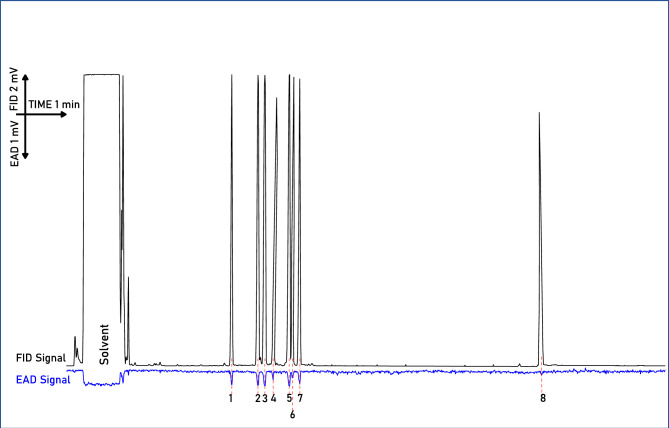
Antennal response of *A. verbasci* adults to the synthetic equivalent (100 NG/µl each) of the 8 antennally active compounds, shown in FID chromatogram (black trace) detected from the headspace of *A. podagraria* umbels during gas chromatography coupled electroantennography. EAD signal (blue trace) contains the antennal responses marked with arabic numbers from 1–8. (1: *α*-pinene; 2: *β*-pinene; 3: *β*-myrcene; 4: *α*-phellandrene; 5: limonene; 6: (*Z*)-*β*-ocimene; 7: (*E*)-*β*-ocimene; 8: germacrene-D).

Regardless of using headspace samples from intact or cut *A. podagraria* florets, the antennae of *A. verbasci* gave robust signals to eight compounds consistently from the 135–144 detected compounds (Fig. 5). These antennally active compounds were *α*-pinene (80–56-8), *β*-pinene (127–91-3), *β*-myrcene (123–35-3), *α*-phellandrene (99–83-2), limonene (138–86-3), (*Z*)-*β*-ocimene (3338–55-4), (*E*)-*β*-ocimene (3779–61-1) and germacrene-D (23,986–74-5). All of these compounds were terpenoids, germacrene-D being sesquiterpenoid while other active compounds were monoterpenoids, and the largest signals were given to *α-*pinene and limonene (Fig. 6). All antennally active compounds were tested with its synthetic equivalents (Fig. 7).

### Behaviour experiments

#### I. Y-Tube behaviour experiments

**Fig. 8:**
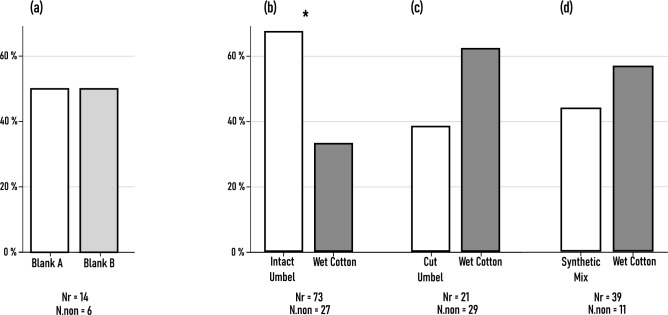
Observed distribution of responding (Nr) *A. verbasci* adults during Y-tube behavioural assays. (**a**): The observed distribution of adults in a blank vs. blank scenario to verify the unbiasedness of the experimental setup. The number of non responding specimens (N.non) are shown under each experimental trial. Asterisk shows significant difference between expected distribution (1:1) and observed distribution based on *Chi-square* test. (**b**–**d**): Experiments involving volatile compounds emanating from intact and cut umbels of *A. podagraria*, and the synthetic mixture of EAD active compounds.

I/(1) During the testing of the experimental setup, we did not detect a difference in choice the beetles made between the two blank arms of the olfactometer. Based on the Chi-square test, the expected distribution did not differ significantly from the observed distribution [X^2^_(1)_ = 0.0 *p* = 1, N = 14]. This indicated that there wasn’t any external factor in the setup that could have influenced the choice of *A. verbasci* adults, ensuring that the setup was reliable for use in other experiments. From the 20 tested specimens, 6 adults (30%) did not make a choice and remained at the starting position (Fig. 8a).

I/(2) During the second set of experiments, adult *A. verbasci* significantly chose intact umbels [X^2^_(1)_ = 8.562 *p* = 0.003, N = 73]. From the 73 responding specimens, 49 (~ 67%) adults selected *A. podagraria* florets, and 24 (~ 33%) wet cotton. From the total 100 tested insects, only 27 did not respond at all (Fig. 8b).

I/(3) In contrast, the preference of adults shifted when umbels with freshly cut stems were presented. Although not significant, adults chose wet cotton more (~ 62%), and only a smaller percent preferred cut umbels (~ 38%) [X^2^_(1)_ = 1.190 *p* = 0.275, N = 21]. As compared to the blank–blank (1) and intact umbel–wet cotton (2) experimental settings, the number of non-responding specimens grew notably. The proportion of non responders were around 58%, almost double what we have previously detected (27–30%), meaning a possible unidentified factor had a negative effect on the insects’ tendency to start (Fig. 8c).

I/(4) In this set of experiments, the synthetic mixture of antennally active compounds detected in GC-EAD recordings were tested. Statistically not significant, yet adults chose wet cotton in greater numbers (22) as opposed to the synthetic mixture (17) [X^2^_(1)_ = 0.641 *p* = 0.423, N = 39]. From the 50 tested specimens, 11 did not move from the starting position (Fig. 8d).

#### II. Petri dish experiments

##### Response latency

**Fig. 9:**
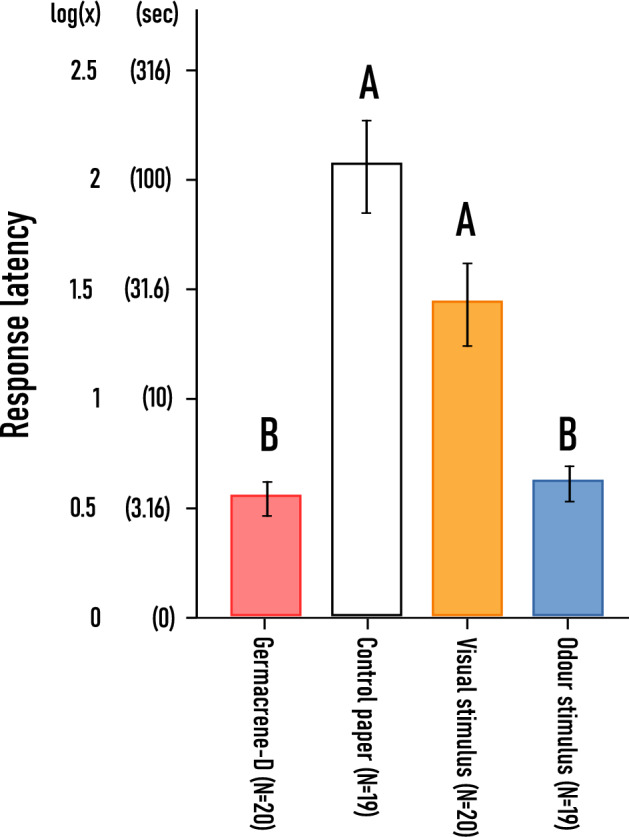
Average time it took for *A. verbasci* to give any form of behavioural response to the introduced stimuli (response latency) (± SE). The results derive from the log(x) transformed data. For clarity, values in seconds are also displayed. Statistically different groups are indicated with capital letters revealed by *Games–Howell* post hoc test (α = 0.05) ‘visual stimulus’ represents the dried umbellet, while ‘odour stimulus’ the headspace sample of *A. podagraria.*

In these sets of experiments we measured how much time passed until the beetle gave a first reaction to the stimulus placed inside the arena. A significant difference was detected between the experiments (F(3.74) = 21.418 (*p* < 0.001)). The shortest time for beetles to give a reaction was for germacrene-D baited discs and to odour stimulus. Both of these trials significantly differed from the control paper and visual stimulus (germacrene-D—control paper: *p* < 0.001; germacrene-D—visual stimulus: *p* < 0.001; odour stimulus—control paper: *p* < 0.001; odour stimulus—visual stimulus: *p* = 0.003), however did not differ from each other (germacrene-D—odour stimulus: *p* = 0.935). The longest latency was induced when only control filter paper was introduced to the insects but this result was not significantly longer than visual stimulus (*p* = 0.147) (Fig. 9).

##### Directional change compared to stimulus

**Fig. 10:**
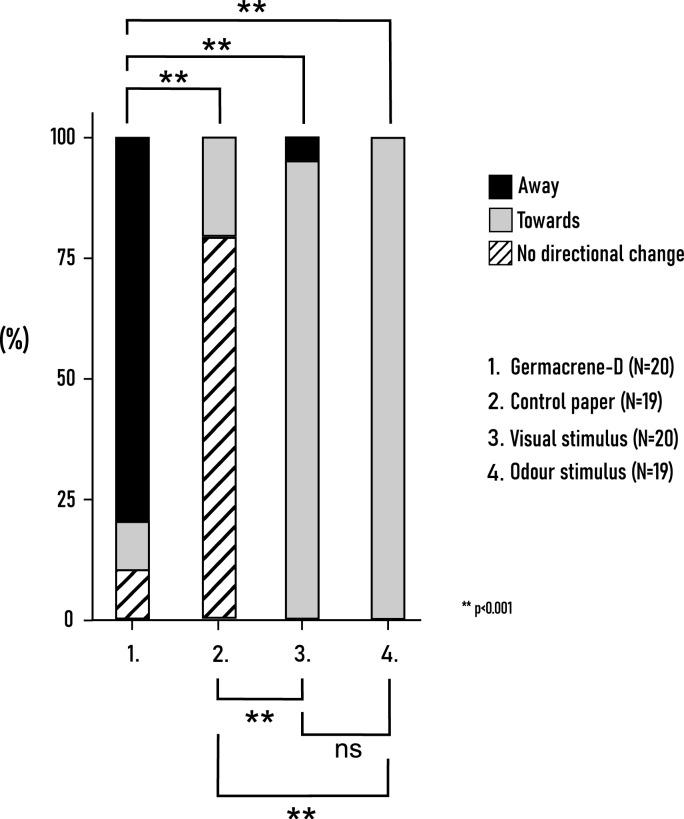
Distribution of incidents within and between experiments given by *A. verbasci*. The possible reaction of adults could vary between moving towards or away from the introduced stimulus or not changing direction at all. ‘Visual stimulus’ represents the dried umbellet, while ‘Odour stimulus’ the headspace sample of *A. podagraria.* Significant differences between distribution of trials were marked with asterisks and were revealed by *Chi-square* test of independence (α = 0.05).

In these experiments, we recorded how specific stimuli influenced the locomotion trajectories of *A. verbasci*. Following stimulus introduction, beetles either moved toward the source, moved away from it, or maintained their original path. Based on the *Chi-square* test, there were significant differences in directional distribution between experiments with a very strong association [X^2^_(6)_ = 103.74 *p* < 0.001, N = 78; Cramer’s V = 0.815]. When germacrene-D was placed near the insects, a significant number of individuals changed their direction and moved away from the stimulus [X^2^_(2)_ = 19.6 *p* < 0.001, N = 20]. When *A. podagraria* headspace sample was dispensed on the filter paper, every beetle (N = 19) chose to go towards the odour stimulus. A significant number of insects similarly changed their direction towards the visual stimulus (*p* < 0.001, N = 20). When control filter paper was placed in, most beetles did not change their path and continued in the direction where they were facing (*p* = 0.19, N = 19). Multiple Chi-square tests were performed to distinguish the distributional differences between pairwise cases (Table S3) (Fig. 10):

##### Stay duration on stimulus

**Fig. 11:**
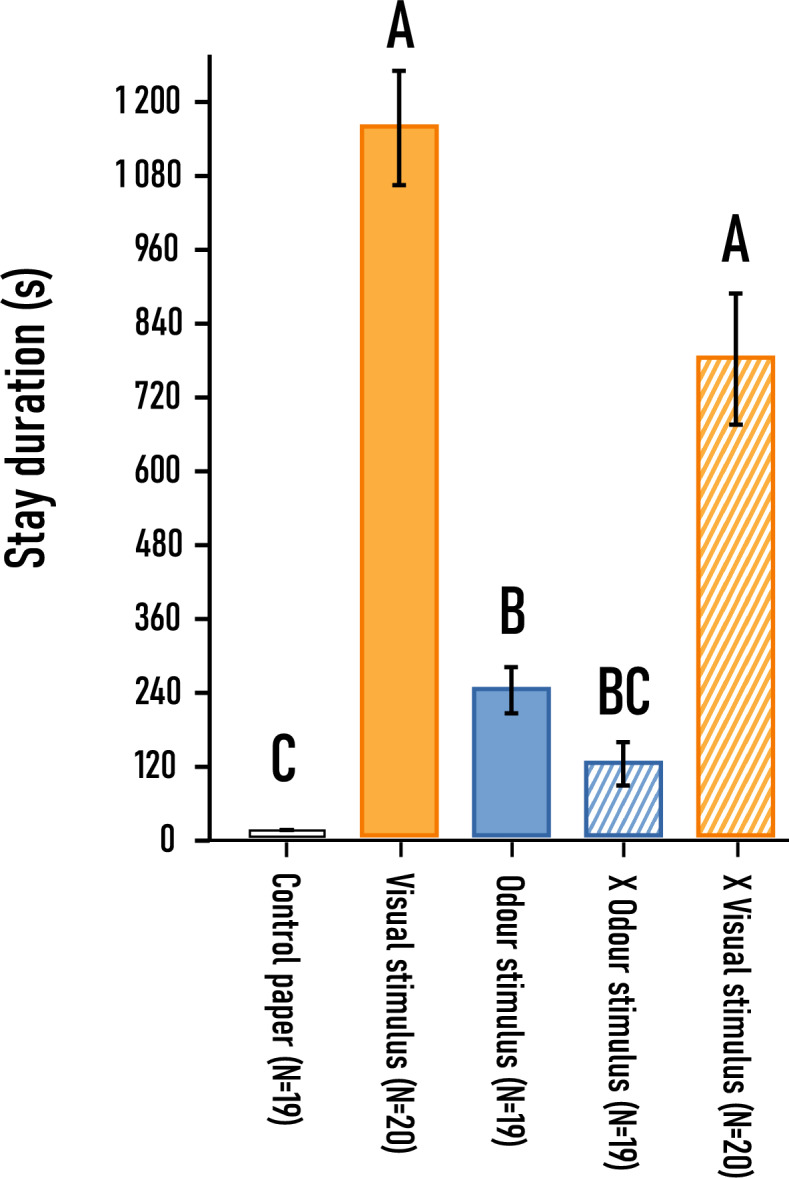
Stay duration of *A. verbasci* adults on different stimuli (± SE). Capital letters indicate statistically significant groups according to *Dunn’s *post hoc test (α = 0.05). In the legend ‘visual stimulus’ represents the dried umbellet, while ‘odour stimulus’ the headspace sample of *A. podagraria;* “x odor stimulus” and “x visual stimulus” indicates when these two stimuli were presented to the beetles at the same time.

There was a significant difference in time spent on filter papers depending on the experimental trial indicated by *Kruskal–Wallis* test [H(4) = 57.85, *p* < 0.001, N = 98]. The longest time was spent on visual stimulus, almost reaching 1200 s on average. A similar pattern was found, when visual stimulus was compared with odour stimulus simultaneously (visual x odour stimulus). It is important to note, that if beetles found the visual stimulus, then many participants did not leave it even after experimental time ended. By its own, beetles stayed an average of ~ 230 s on odour stimulus and this duration was not significantly different, when it was paired against visual stimulus simultaneously. The shortest time was spent on control paper discs, on average the insects stayed on it ~ 10 s before leaving it (Fig. 11). By *Dunn’s *Post Hoc test, significant differences were revealed between groups (Table S).

##### First stimulus found and final decision

**Fig. 12:**
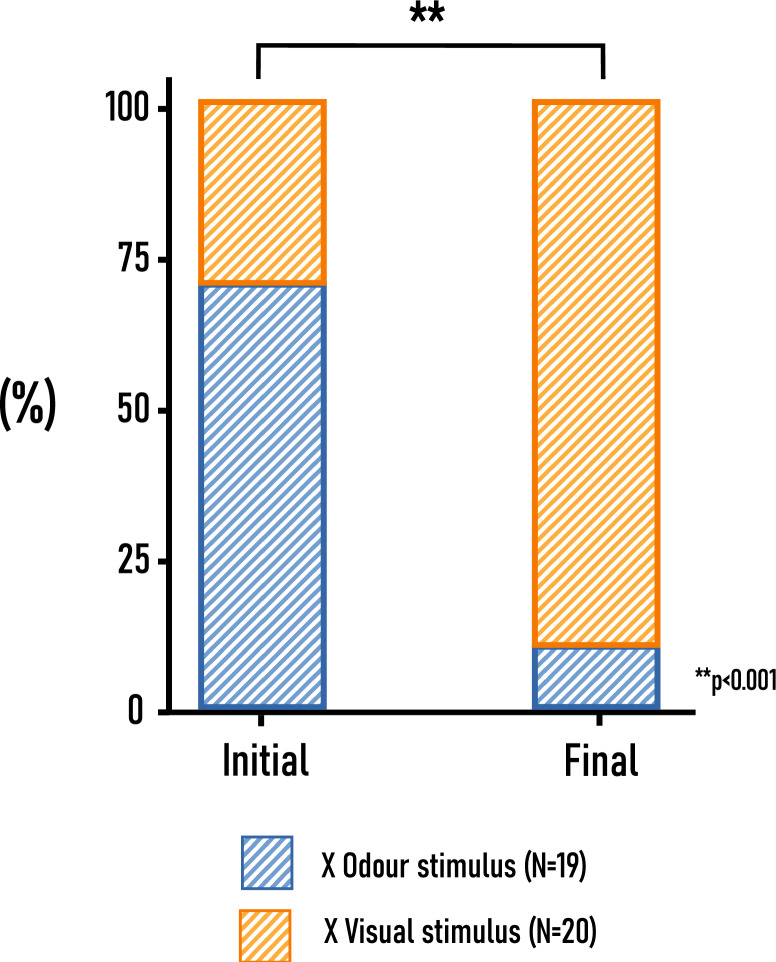
Distribution of insects according to their initial choice and the final distribution at the end of the experiment. *One-Sample Binomial* test revealed that the distribution of initial choice did not differ from the hypothetical 1:1; while *Chi-square* test of independence revealed significant difference between the initial and final distribution of insects (α = 0.05). In the legend ‘visual stimulus’ represents the dried umbellet, while ‘odour stimulus’ the headspace sample of *A. podagraria;* “x odor stimulus” and “x visual stimulus” indicates when these two stimuli were presented to the beetles at the same time.

During the simultaneous comparison of odour and visual stimulus more insects chose the odour stimulus first (70%), however this was not significantly greater than the expected distribution of 1:1 indicated by the *One-Sample Binomial* test (*p* = 0.115, N = 20). When comparing the distribution of the first (14:6) and final choice (2:18), Chi-square test revealed a significant difference [X^2^_(1)_ = 15 *p* < 0.001, N = 40; Cramer’s V = 0.612]. At the end of the experiment the initial choice shifted towards the visual one (Fig. 12).

##### Duration of 3 cm locomotion

The duration at which beetles moved 3 cm from the germacrene-D treated papers (~ 20,3 s) did not differ significantly from how insects moved in an empty arena (~ 18,7 s), revealed by *Mann–Whitney U* test (U = 165, z =  − 0.44, *p* = 0.675, N = 38).

## Discussion

The larvae of *A. verbasci* are known to cause serious damage on wool, feathers, dried plant matter, leather, fur, household items, etc.^33,34^, while little attention is paid to the host breadth of adult insects. The presence of *A. verbasci* on *A. podagraria* have been described^24^, however the possible cause for this interaction have not been well studied before.

There is scarce information about the chemical ecology of this species, however it is known that females emit sex pheromone during mate calling^35^. Apart from sex pheromones, there is only one notable compound that elicited mass attraction in *A. verbasci*. Originally implemented in a *Chrysanthemum* experiment for trapping flower thrips^36^, it was observed that *p*-anisaldehyde baited traps caught adults of *A. verbasci*; moreover it lured both sexes^23^. In accordance with the literature, we did not identify *p*-anisaldehyde in the headspace of *A. podagraria*^37–39^, yet it elicited an attraction in our experiments. Volatile components in flowers play a crucial role in attracting insects and influencing interactions with their environment. In the headspace volatile, we have detected compounds that are common floral volatiles that are characteristic for the Apiaceae family (Table S1). If we examine the antennally active floral volatiles (Fig. 6), the compounds appear to be more general across plant species rather than specific to Apiaceae. In general, adult *A. verbasci* and other carpet beetles prefer umbelliferous plants, yet can be observed on other plant families like Asteraceae^24^. Furthermore, it should be mentioned that in the context of host plant selection, the relative proportions of behaviorally active volatiles are more determining than their mere presence in the host plant. Herbivorous insects are adept at detecting and responding to a complex blend of plant volatiles, which provide essential information on plant identity and quality. Insects analyse these complex mixtures for host recognition, suggesting that the ability to discern ratios of volatiles is crucial for effective host selection^40^. All plants emit volatiles, the challenge lies in detecting reliable cues that indicate plant quality, further underscoring the importance of the ratio of volatiles over mere composition^41^. Even small modulations in ratio can be detected by insects, which can be a determining factor in evaluating host plant condition^42^ and distinguishing between non-host and host plants based on ratios^43^. The active volatiles detected during electrophysiology were all mono- and sesquiterpenes, which in itself does not exclude the possibility of detecting other chemical groups and volatiles. Yet based on the host plants of *A. Verbasci—*which are mainly umbelliferous plants that contain an abundant part of terpenes in their chemical composition—it is possible that *A. verbasci* has a terpene-tuned olfactory receptor array.

According to the heatmap (Fig. 6), the strongest antennal responses were elicited by *α*-pinene, *β*-pinene, *β*-myrcene and limonene. In many Coleoptera, especially wood- and bark-associated species, the same volatiles function as host-kairomones or as synergists of pheromones^44^. For Anthrenus beetles, the detection of these monoterpenes could signal possible foraging sites as adults feed on pollen and nectar^3^ and these compounds are commonly found in many Apiaceae species^37^. Although in trace amounts, 4,8-Dimethyl-1,3,7-nonatriene—or DMNT—has also been identified, which is an important compound regarding plant–insect interaction. DMNT emission is strongly associated with insect herbivory, the accumulation of this compound can further induce plant defence by the production of protease inhibitors that render the plant less palatable to herbivores^45,46^. However, during GC-EAD the antennae did not respond to DMNT when testing cut umbel volatiles. It must be underscored that the relative area of DMNT in the collected headspace was around 0.01%, which may be under the detectable threshold of the antennae. Another answer could be that during antennal preparation for EAD experiments, the DMNT sensitive sensilla were covered by the capillary during EAD thus no response was given to this compound.

Y-tube experiments revealed that olfactory cues alone were sufficient enough to elicit attraction. Similarly in Petri dish experiments, discs containing the headspace volatile of intact *A. podagraria* flowers initiated a rapid attraction when presenting the stimuli. However this behaviour was only observable, when intact umbels were used in experiments, suggesting that due to damage, additional volatiles were released which caused a possible aversion behaviour in insects. The positive response of adults decreased when volatiles came from cut umbels. In Y-tube assays, more adults chose wet cotton pads over cut umbel volatiles. Notably, the number of non-responding specimens increased sharply compared to other trials. In the blank vs. blank scenario, 30% of adults were non-responsive, suggesting a baseline non-response rate of ~ 30% in this *A. verbasci* population. This remained similar in other trials but rose to 58% when cut umbels were used (Fig. 8). This could mean that there was an extra factor that withheld insects from leaving the starting position, which resulted in an almost double the observed non respondent percentage. If we compare the ratio of antennally active compounds in the volatilome of intact and cut umbels we can see that most of the compounds have increased slightly. The only compound which drastically increased was germacrene-D. In the volatilome of cut umbels the relative area of germacrene-D was almost 15%, yet in the intact umbels it was around 3%; a five times difference in relative area. Germacrene-D is commonly found in various plant species, and has significant effects on insect behavior and physiology. This compound is primarily recognised for its role in plant defense mechanisms against herbivores, acting as a repellent or deterrent to various insect species. Germacrene-D exhibits a repellent effect on ticks and mites^47,48^, while it can attract beneficial insects such as pollinators, while simultaneously deterring herbivorous pests^49,50^. Furthermore, germacrene-D may serve as a cue for predators, thus affecting the dynamics of plant–insect interactions^51^. The elevated amount of germacrene-D could have been an important cue for *A. verbasci* adults, signaling that the host plant quality is altered, and not sufficient for foraging. The behaviour importance of germacrene-D is further supported by the observations made in Petri dish experiments: Response latency to germacrene-D stimulus was the shortest, meaning the insect perceived a change in its environment that forced out a rapid action. Behavioral assays clearly demonstrated that germacrene-D elicits an aversion effect, beetles oriented away from the source. The combination of this unique behavioral pattern and the short response latency strongly supports the conclusion that germacrene-D acts as a repellent. Such directed avoidance behavior is consistent with observations in other insect species reacting to repellent substances^52^. Given that the adult insects have an approximate body length of 3 mm^53^, the 3 cm distance represents a significant displacement, roughly ten times their body size.

For herbivorous insects, vision plays a more significant role at greater distances, whereas olfaction becomes more influential at closer proximities^2^. The prevailing view suggests that floral visual cues play the dominant role for *A. verbasci*^33^, Imamura even suggesting that floral scent is likely irrelevant^34^. Petri dish experiments revealed that response latency to visual stimuli was not significantly shorter than control filter paper disc treatments. In the case of control, this could suggest that the white coloration of the control disc may have mimicked some floral cues, despite disparities in shape and detailed features, and the presence of yellow and green hues within the actual flowers. It is possible that from the distance experienced by the beetles, they could not distinguish the background contrast well enough from the control paper and the visual stimulus. Additionally, the vibrant colour of flowers were diminished due to the drying process, a necessary step to ensure that visual stimuli did not emit any odour at all. Furthermore, adult insects, particularly those in flight, primarily perceive visual stimuli from various angles in their natural environment. When comparing the response latency to visual and odour stimulus, a significant difference was observed. It is possible that the visual cue was outside the effective visual field of the insect. If the visual target starts outside the frontal acute zone or in a region with poor acceptance angles, the insect must compensate for this first^54^. The perception of visual stimuli on the largely two-dimensional arena surface may have been compromised. These factors together could have influenced the choice of beetles during experiments. This discrepancy in perception conditions may account for the statistically significant difference observed between response latency to olfactory and visual stimuli. It is possible, that in nature, the visual stimuli created by the large patches of white umbels contrasting to the background are more perceivable from a distance for *A. verbasci,* yet adults rely primarily on olfactory cues to assess and select the most suitable flower at a close range, as chemical signals provide critical information about nectar availability, floral quality, and host suitability. It is important to note that in certain species, such as *Rhagoletis pomonella*, both visual and olfactory cues can induce approach behaviors, with the stronger stimulus dictating the response^55^.

A significant number of adults shifted their direction towards the visual stimuli. Overall, in the context of the experimental design, both olfactory and visual stimuli (excluding the repellent germacrene-D) elicited an approach behavior. The animals consistently oriented towards both types of stimuli, with no significant difference between the two. This indicates that, based on these findings, both visual and olfactory cues are individually attractive and effective for the adult insects. This phenomenon is not unique; for example, the Asian longhorn beetle (*Anoplophora glabripennis*) demonstrates host plant recognition (specifically *Acer negundo*) through both visual and olfactory stimuli, whether presented separately or in combination^56^. Collectively, our results suggest that both the odor and the overall properties of visual signal of flowers play a crucial role in foraging behavior. It must be underlined, that the visual stimuli used in these sets of experiments differed from natural ones due to the drying process.

When presented with a simultaneous choice between visual and olfactory stimuli, the insects initially selected the disc with headspace volatiles of *A. podagraria*. This initial choice suggests a predominant role for olfactory cues in host plant recognition. However, it’s crucial to acknowledge the potential limitations in visual stimulus perception within the experimental setup, as discussed previously. A notable observation was that a significant proportion of animals subsequently departed the scented disc and migrated to the flower disc (Fig. 12). This suggests that olfactory cues exert a stronger initial influence in this experimental setup. It is worth noting—although not significant—that there was an observably prolonged presence of insects on scented paper discs when they were presented by itself, and beetles departed from these baited discs earlier, when visual stimulus was also available (Fig. 9). The results indicate that beetles initially oriented to the odour cue, but in the absence of a physical reward, most shifted to the visual stimulus. The exploration of both odor and visual stimuli by most insects could reflect to a degree of behavioral flexibility.

We analysed the time spent on stimulus, the shortest time was consistently recorded on the control paper. This was significantly different from all other stimuli, with the exception when odour stimulus was compared with visual one together (Fig. 9). Even though this was not significant, in reality this meant that the average time spent on the control was ~ 10 s compared to the average of odour stimulus ~ 120 (s). Conversely, residence time was longest on visual stimulus, beetles often stayed there until the end of the experiment. Beyond mere duration, distinct behavioral patterns were noted. On the control paper, insects exhibited continuous movement, frequently attempting to leave the disc upon reaching its edge. In contrast, on visual stimulus beetles crawled on the flower, displaying exploratory movements, initiating tactile behaviour at first. This behaviour stopped and instead the beetles moved into the flower structure and remained stationary until the end of the experiment, regardless of whether the visual stimulus was by itself, or paired against odour stimulus, and regardless of which was discovered first. On scented discs, insects showed searching behaviour, many touching the surface of discs with their head. This is relevant, since the maxillary palpi is an important organ for evaluating food source quality^57^. No significant difference was found in residence times on discs containing the same stimuli, irrespective of whether those stimuli were presented in isolation or in combination with other stimuli in the arena. The physical complexity of the visual stimulus may explain why the insects spent the most time interacting with it. This effect could be due to the additional tactile cues provided by the stimulus compared to the texture of filter papers, as well as the potential refuge offered by the dried flower.

In summary, apart from visual signals, volatile compounds alone play a determining factor when it comes to host recognition in *A. verbasci.* We first report the physiologically active volatiles of *A. podagraria* detected by *A. verbasci*. However, the specific compounds eliciting positive behavioural responses could not be identified; the tested 1:1 ratio synthetic blend did not attract adults. It was proven in Y-tube and Petri dish bioassays that olfactory cues alone are enough to elicit an attraction. This attraction was negated if odour was originating from a damaged floral umbel, suggesting that there are certain volatiles that increase avoidance in adults. The experiments with damaged umbels showed that adults avoided germacrene-D and quickly moved to distant positions, suggesting that this compound is repellent. It is strongly indicated that elevated compound levels of germacrene-D can hinder the explorative behaviour of adults. Furthermore it was determined that adults initially located flower scented odour sources, but later almost all insects migrated to visual stimuli highlighting the importance of olfaction and vision in searching behaviour.

## Supplementary Information

Supplementary Information.

## Data Availability

Raw data of experiments and recordings have been uploaded and publicly available at figshare: (https://figshare.com/s/b983bf3b153a371f783d).
