# Supplementary material for: The interplay of olfaction and vision in host plant selection by Anthrenus verbasci
Source: Sci Rep. 2025 Oct 27;15:37341. doi: 10.1038/s41598-025-22240-7 (PMC12559408; doi:10.1038/s41598-025-22240-7)

Supplementary Information for the manuscript titled:

The Interplay of Olfaction and Vision in Host Plant Selection by *Anthrenus verbasci*

Authors and affiliations:

FERENC DEUTSCH and SÁNDOR KECSKEMÉTI,

Department of Chemical Ecology, Plant Protection Institute, Centre for Agricultural Research, HUN-REN, Budapest, Hungary

**Corresponding author: kecskemeti.sandor@atk.hun-ren.hu*

ORCID information:

Ferenc Deutsch: 0000-0002-7625-6227

Sándor Kecskeméti: 0000-0002-3952-8174

Table S1: List of compounds detected and identified in the headspace sample of intact and cut *A. podagraria* umbels using GC-MS. Detected compounds are in ascending order based on calculated Kovats retention indices (Ri_a) and NIST library records (RI_b).

#	RI_a	RI_b	RT	Compound Names	CAS	Relative area of Aegopodes podagraria	
						**Cut Umbels**	**Intact Umbels**
**1.**		761	2.99	unknown compound		0	0.01 ± 0.00
**2.**		768	3.08	sec-butyl acetate	105-46-4	0	0.02 ± 0.00
**3.**		773	3.14	unknown compound		0	0.01 ± 0.00
**4.**	770	777	3.19	toluene	108-88-3	0.03 ± 0.00	0.05 ± 0.00
**5.**	784	788	3.34	3-hexanone	589-38-8	0.11 ± 0.01	0.05 ± 0.00
**6.**	788	791	3.38	2-hexanone	591-78-6	0.12 ± 0.02	0.04 ± 0.00
**7.**	797	795	3.44	3-hexanol	623-37-0	0.12 ± 0.01	0.02 ± 0.00
**8.**	803	800	3.49	2-hexanol	626-93-7	0.16 ± 0.01	0.07 ± 0.01
**9.**		815	3.69	siloxane residue		0.05 ± 0.00	0.02 ± 0.00
**10.**		819	3.74	siloxane residue		0.24 ± 0.05	0.01 ± 0.00
**11.**		820	3.76	siloxane residue		0.07 ± 0.01	0
**12.**	855	854	4.2	3-hexen-1-ol	544-12-7	0.02 ± 0.00	0.01 ± 0.00
**13.**		862	4.3	unknown compound		0.02 ± 0.00	0.01 ± 0.00
**14.**	867	865	4.34	1-hexanol	111-27-3	0.02 ± 0.00	0.01 ± 0.00
**15.**	866	870	4.41	*p*-xylene	106-42-3	0.02 ± 0.00	0.02 ± 0.00
**16.**		872	4.44	unknown compound		0.01 ± 0.00	0
**17.**		874	4.46	unknown compound		0	0.01 ± 0.00
**18.**		883	4.58	unknown compound		0	0.01 ± 0.00
**19.**	902	902	4.82	heptanal	111-71-7	0.01 ± 0.00	0.01 ± 0.00
**20.**	926	926	5.18	tricyclene	508-32-7	0.02 ± 0.00	0.03 ± 0.00
**21.**	920	929	5.23	*β*-thujene	28634-89-1	1.54 ± 0.15	1.42 ± 0.15
**22.**	939	937	5.35	*α*-pinene	80-56-8	8.27 ± 0.45	9.29 ± 0.62
**23.**	955	953	5.58	camphene	79-92-5	0.39 ± 0.02	0.32 ± 0.01
**24.**		958	5.66	unknown compound		0	0.02 ± 0.00
**25.**	960	963	5.74	benzaldehyde	100-52-7	0.02 ± 0.00	0.01 ± 0.00
**26.**	977	977	5.94	sabinene	3387-41-5	2.58 ± 0.09	2.52 ± 0.27
**27.**	980	982	6.02	*β*-pinene	18172-67-3	15.27 ± 1.22	16.28 ± 1.89
**28.**	993	992	6.16	*β*-myrcene	123-35-3	8.93 ± 0.69	7.15 ± 0.86
**29.**	1001	1003	6.33	octanal	124-13-0	0.04 ± 0.01	0.06 ± 0.00
**30.**	1012	1008	6.4	*α*-phellandrene	99-83-2	1.06 ± 0.04	0.73 ± 0.06
**31.**	1013	1014	6.5	3-carene	13466-78-9	0.01 ± 0.00	0.01 ± 0.00
**32.**	1019	1020	6.59	*α*-terpinene	99-86-5	0.14 ± 0.02	0.05 ± 0.01
**33.**	1024	1028	6.71	*p*-cymene	99-87-6	0.16 ± 0.01	0.08 ± 0.01
**34.**	1032	1034	6.8	limonene	5989-27-5	25.92 ± 1.01	17.81 ± 1.31
**35.**	1041	1038	6.87	(*Z*)-*β*-ocimene	3338-55-4	2.65 ± 0.04	1.72 ± 0.09
**36.**	1044	1046	7	benzeneacetaldehyde	122-78-1	0.16 ± 0.02	0
**37.**	1052	1049	7.04	(*E*)-β-ocimene	3779-61-1	0.85 ± 0.10	0.53 ± 0.03
**38.**		1058	7.17	unknown compound		0.02 ± 0.00	0.01 ± 0.00
**39.**	1062	1062	7.24	γ-terpinene	99-85-4	0.23 ± 0.02	0.10 ± 0.01
**40.**		1070	7.37	unknown compound		0.01 ± 0.00	0
**41.**	1089	1092	7.7	terpinolene	586-62-9	0.17 ± 0.01	0.06 ± 0.00
**42.**	1099	1100	7.82	linalool	78-70-6	0.10 ± 0.01	0.04 ± 0.00
**43.**	1104	1105	7.89	nonanal	124-19-6	0.04 ± 0.01	0.02 ± 0.00
**44.**	1110	1110	7.98	1-octen-3-yl-acetate	198242	0.07 ± 0.00	0
**45.**	1114	1117	8.07	4,8-dimethyl-1,3,7-nonatriene (DMNT)	51911-82-1	0.01 ± 0.00	0
**46.**	1124	1123	8.16	3-octanol, acetate	4864-61-3	0.02 ± 0.00	0.01 ± 0.00
**47.**	1131	1131	8.28	(4*E*,6*Z*)-allo-ocimene	7216-56-0	0.05 ± 0.00	0.02 ± 0.00
**48.**		1138	8.39	unknown compound		0.01 ± 0.00	0
**49.**	1147	1146	8.5	2,6-dimethyl-1,3,5,7-octatetraene		0.02 ± 0.00	0.01 ± 0.00

**50.**		1152	8.6	siloxane residue		0.02 ± 0.00	0.01 ± 0.00
**51.**	1154	1155	8.64	lilac aldehyde B	53447-45-3	0.01 ± 0.00	0
**52.**	1168	1168	8.84	benzaldehyde, 3-ethyl-	34246-54-3	0.13 ± 0.01	0
**53.**	1206	1184	9.07	benzaldehyde, 4-ethyl-	4748-78-1	0.06 ± 0.01	0
**54.**		1191	9.18	unknown compound		0.02 ± 0.00	0.01 ± 0.00
**55.**	1200	1199	9.29	dodecane		0.01 ± 0.00	0.01 ± 0.00
**56.**		1207	9.4	unknown compound		0.03 ± 0.00	0.02 ± 0.00
**57.**	1259	1257	10.11	linalool acetate	115-95-7	0.18 ± 0.01	0.06 ± 0.00
**58.**		1263	10.19	unknown compound		0.03 ± 0.00	0.01 ± 0.00
**59.**		1269	10.28	unknown compound		0.09 ± 0.01	0
**60.**		1281	10.45	unknown compound		0.02 ± 0.00	0.01 ± 0.00
**61.**	1281	1289	10.56	ethanone, 1-(4-ethylphenyl)-	937-30-4	0.04 ± 0.00	0
**62.**	1286	1292	10.61	bornyl acetate	76-49-3	0.23 ± 0.01	0.06 ± 0.00
**63.**	1300	1300	10.71	tridecane	629-50-5	0.06 ± 0.01	0.04 ± 0.00
**64.**	1300	1306	10.8	trans-pinocarvyl acetate	1686-15-3	0.05 ± 0.00	0.01 ± 0.00
**65.**		1321	11	unknown compound		0.12 ± 0.01	0
**66.**		1325	11.05	siloxane residue		0.05 ± 0.01	0
**67.**		1326	11.07	unknown compound		0	0.01 ± 0.00
**68.**		1337	11.21	unknown compound		0.01 ± 0.00	0
**69.**		1347	11.34	elemen isomer		0.09 ± 0.01	0.02 ± 0.00
**70.**	1345	1359	11.5	*α*-cubebene	17699-14-8	0.02 ± 0.00	0
**71.**		1364	11.58	unknown compound		0.06 ± 0.00	0
**72.**	1373	1381	11.8	cyclosativene	22469-52-9	0.03 ± 0.00	0.01 ± 0.00
**73.**	1373	1383	11.83	ylangene	14912-44-8	0.01 ± 0.00	0
**74.**	1377	1387	11.88	copaene	3856-25-5	0.19 ± 0.01	0.07 ± 0.01
**75.**	1389	1392	11.94	1-tetradecene	1120-36-1	0.04 ± 0.00	0.01 ± 0.00
**76.**		1394	11.98	unknown compound		0.02 ± 0.00	0
**77.**	1391	1401	12.07	*β*-elemen	515-13-9	0.49 ± 0.06	0.12 ± 0.01
**78.**		1406	12.14	unknown compound		0.02 ± 0.00	0
**79.**		1410	12.17	unknown compound		0.02 ± 0.00	0
**80.**		1411	12.19	unknown compound		0	0.00 ± 0.00
**81.**		1415	12.25	unknown compound		0.01 ± 0.00	0
**82.**		1423	12.35	unknown compound		0.01 ± 0.00	0.00 ± 0.00
**83.**	1420	1435	12.49	*β*-caryophyllene	87-44-5	0.41 ± 0.05	0.10 ± 0.01
**84.**	1430	1443	12.6	*β*-copaene	18252-44-3	0.14 ± 0.02	0.03 ± 0.00
**85.**	1432	1448	12.66	*β*-gurjurene	17334-55-3	0.04 ± 0.01	0.01 ± 0.00
**86.**		1451	12.7	unknown compound		0.02 ± 0.00	0
**87.**	1455	1460	12.8	*(Z)-β*-farnesene	28973-97-9	2.40 ± 0.22	0.78 ± 0.09
**88.**	1456	1469	12.92	*α*-caryophyllene	6753-98-6	0.63 ± 0.03	0.20 ± 0.02
**89.**	1478	1478	13.03	(Z)-muurola-4(15),5-diene	157477-72-0	0.13 ± 0.01	0.02 ± 0.00
**90.**	1479	1489	13.17	*γ*-muurolene	30021-74-0	0.12 ± 0.01	0.01 ± 0.00
**91.**	1485	1496	13.27	germacrene D	23986-74-5	14.94 ± 0.16	2.91 ± 0.42
**92.**	1507	1511	13.45	*α*-farnesene	502-61-4	7.14 ± 0.42	1.35 ± 0.09
**93.**		1521	13.56	unknown compound		0.02 ± 0.00	0.01 ± 0.00
**94.**	1514	1528	13.64	*γ*-cadinene	39029-41-9	0.14 ± 0.01	0.01 ± 0.00
**95.**	1524	1535	13.73	*δ*-cadinene	483-76-1	0.10 ± 0.01	0.01 ± 0.00
**96.**	1540	1545	13.86	*α*-cadinene	17627-25-7	0.01 ± 0.00	0
**97.**		1551	13.92	unknown compound		0.03 ± 0.00	
**98.**		1562	14.06	unknown compound		0.01 ± 0.00	0.00 ± 0.00
**99.**		1575	14.21	unknown compound		0.01 ± 0.00	0
**100.**		1582	14.3	unknown compound		0.01 ± 0.00	0.01 ± 0.00
**101.**	1572	1591	14.4	germacren D-4-ol	198991-79-6	0.13 ± 0.01	0.02 ± 0.00
**102.**	1585	1599	14.49	(*E*)-(*Z*)-*α*-bisabolene epoxide		0.13 ± 0.01	0.02 ± 0.00
**103.**	1599	1612	14.64	salvial-4(14)-en-1-one	73809-82-2	0.02 ± 0.00	0
**104.**		1626	14.8	unknown compound		0.01 ± 0.00	0
**105.**		1658	15.16	unknown compound		0.01 ± 0.00	0
**106.**		1668	15.27	unknown compound		0.02 ± 0.00	0

**107.**		1691	15.53	unknown compound		0.02 ± 0.00	0
**108.**	1700	1698	15.62	heptadecane		0.02 ± 0.00	0
**109.**		1715	15.8	unknown compound		0.09 ± 0.01	0
**110.**		1727	15.94	unknown compound		0.02 ± 0.00	0
**111.**	1728	1733	16	2,6-diisopropylnaphthalene	24157-81-1	0	0.00 ± 0.00
**112.**		1737	16.08	unknown compound		0	0.01 ± 0.00
**113.**		1755	16.23	unknown compound		0.01 ± 0.00	0
**114.**		1762	16.3	unknown compound		0.03 ± 0.00	0
**115.**		1798	16.7	unknown compound		0.01 ± 0.00	0.01 ± 0.00
**116.**		1800	17.59	unknown compound		0	0.01 ± 0.00
**117.**		1802	17.64	unknown compound		0	0.04 ± 0.00
**118.**		1805	17.77	unknown compound		0.02 ± 0.00	0
**119.**		1834	17.06	unknown compound		0.01 ± 0.00	0
**120.**		1845	17.17	unknown compound		0.01 ± 0.00	0
**121.**		1865	17.38	unknown compound		0.02 ± 0.00	0
**122.**		1899	17.75	unknown compound		0	0.01 ± 0.00
**123.**		1916	17.9	unknown compound		0.16 ± 0.01	0
**124.**		1920	17.93	unknown compound		0	0.01 ± 0.00
**125.**		1931	18.04	unknown compound		0.05 ± 0.00	0
**126.**		1946	18.19	unknown compound		0.03 ± 0.00	0
**127.**		1962	18.35	unknown compound		0.01 ± 0.00	0
**128.**		1968	18.4	unknown compound		0.12 ± 0.02	0
**129.**		1972	18.42	unknown compound		0	0.01 ± 0.00
**130.**		1978	18.5	unknown compound		0.03 ± 0.00	0
**131.**		1985	18.57	unknown compound		0.24 ± 0.02	0
**132.**		1992	18.64	unknown compound		0.01 ± 0.00	0
**133.**		1995	18.74	unknown compound		0.02 ± 0.00	0
**134.**			18.79	unknown compound		0.02 ± 0.00	0
**135.**			18.84	unknown compound		0.04 ± 0.00	0
**136.**			18.91	unknown compound		0.02 ± 0.00	0
**137.**			19.04	unknown compound		0.01 ± 0.00	0
**138.**			19.09	unknown compound		0.02 ± 0.00	0.02 ± 0.00
**139.**			19.19	unknown compound		0.01 ± 0.00	0
**140.**			19.24	unknown compound		0	0.03 ± 0.00
**141.**			19.33	unknown compound		0.04 ± 0.00	0
**142.**			19.39	unknown compound		0.01 ± 0.00	0
**143.**			19.64	unknown compound		0	0.02 ± 0.00
**144.**			19.8	unknown compound		0.16 ± 0.01	
**145.**			20.02	unknown compound			0.01 ± 0.00
**146.**			20.27	unknown compound			0.01 ± 0.00
**147.**			20.21	unknown compound		0.01 ± 0.00	0
**148.**			20.34	unknown compound		0	0.05 ± 0.01
**149.**			20.36	unknown compound		0	0.09 ± 0.00
**150.**			20.45	unknown compound		0.01 ± 0.00	0
**151.**			20.64	unknown compound		0.01 ± 0.00	0
**152.**			20.8	unknown compound		0	0.01 ± 0.00
**153.**			20.83	unknown compound		0.01 ± 0.00	0.04 ± 0.00
**154.**			20.99	unknown compound		0	0.01 ± 0.00
**155.**			21.04	unknown compound		0	0.02 ± 0.00
**156.**			21.12	unknown compound		0	0.01 ± 0.00
**157.**			21.22	unknown compound		0	0.01 ± 0.00
**158.**			21.31	unknown compound		0	0.01 ± 0.00
**159.**			21.4	unknown compound		0	0.01 ± 0.00
**160.**			21.43	unknown compound		0	0.00 ± 0.00
**161.**			21.56	unknown compound		0.08 ± 0.01	0
**162.**			21.66	unknown compound		0	0.01 ± 0.00
**163.**			21.73	unknown compound		0	0.02 ± 0.00

**164.**	21.82	unknown compound	0	0.13 ± 0.00
**165.**	22.06	unknown compound	0	0.01 ± 0.00
**166.**	22.21	unknown compound	0	0.01 ± 0.00
**167.**	22.33	unknown compound	0	0.01 ± 0.00
**168.**	22.53	unknown compound	0	0.04 ± 0.00
**169.**	22.65	unknown compound	0	0.10 ± 0.01
**170.**	22.85	unknown compound	0	0.00 ± 0.00
**171.**	23.01	unknown compound	0	0.18 ± 0.01
**172.**	23.15	unknown compound	0	0.01 ± 0.00
**173.**	23.17	unknown compound	0.04 ± 0.00	0
**174.**	23.29	unknown compound	0	0.09 ± 0.00
**175.**	23.5	unknown compound	0.02 ± 0.00	0
**176.**	23.53	unknown compound	0	1.17 ± 0.11
**177.**	23.62	unknown compound	0	0.03 ± 0.00
**178.**	23.85	unknown compound	0	0.06 ± 0.00
**179.**	24.11	unknown compound	0	15.92 ± 1.88
**180.**	24.35	unknown compound	0	1.06 ± 0.08
**181.**	24.43	unknown compound	0	0.72 ± 0.02
**182.**	24.81	unknown compound	0	0.72 ± 0.07
**183.**	25.04	unknown compound	0.01 ± 0.00	0
**184.**	25.17	unknown compound	0	3.23 ± 0.23
**185.**	25.5	unknown compound	0	8.33 ± 0.64
**186.**	25.64	unknown compound	0	0.19 ± 0.02
**187.**	25.74	unknown compound	0	0.02 ± 0.00
**188.**	25.86	unknown compound	0	0.04 ± 0.00
**189.**	25.9	unknown compound	0	0.03 ± 0.00
**190.**	26.05	unknown compound	0	0.14 ± 0.01
**191.**	26.43	unknown compound	0	0.94 ± 0.07
**192.**	26.8	unknown compound	0	0.16 ± 0.02
**193.**	27.12	unknown compound	0	0.06 ± 0.00
**194.**	27.34	unknown compound	0	0.06 ± 0.00
**195.**	27.51	unknown compound	0	0.01 ± 0.00
**196.**	27.56	unknown compound	0	0.01 ± 0.00
**197.**	27.82	unknown compound	0	0.26 ± 0.02
**198.**	28.08	unknown compound	0	0.04 ± 0.00
**199.**	28.3	unknown compound	0	0.04 ± 0.00
**200.**	28.75	unknown compound	0	0.19 ± 0.02
**201.**	29.37	unknown compound	0	0.01 ± 0.00
**202.**	29.46	unknown compound	0	0.03 ± 0.00
**203.**	30.13	unknown compound	0	0.13 ± 0.02
**204.**	30.87	unknown compound	0	0.09 ± 0.00
**205.**	31.16	unknown compound	0	0.01 ± 0.00
**206.**	31.51	unknown compound	0	0.10 ± 0.01
**207.**	31.91	unknown compound	0	0.52 ± 0.06
**208.**	32.42	unknown compound	0	0.03 ± 0.00

Table S2: Results of multiple Chi-square tests between experimental trials

Germacrene – Control paper: [$X^2_{(2)}$=26.6 p<0.001, N=39; Cramer's V = 0.826]

Germacrene – Visual stimulus: [$X^2_{(2)}$=28.997 p<0.001, N=40; Cramer's V = 0.851]

Germacrene – Odour stimulus: [$X^2_{(2)}$=31.757 p<0.001, N=39; Cramer's V = 0.902]

Visual stimulus – Control paper: [$X^2_{(2)}$= 25.774 p<0.001, N=39 Cramer's V = 0.813]

Visual stimulus – Odour stimulus: [$X^2_{(1)}$=0.975 p=0.323, N=39; Cramer's V = 0.158]

Odour stimulus – Control paper: [$X^2_{(1)}$=24.783 p<0.001 N=38; Cramer's V = 0.808]

Table S3: Result of Dunn's Post hoc test between tested groups

Control paper – x Odour stimulus:	z = -1.23, p = 1	x Odour stimulus – x Visual stimulus:	z = -3.7, p = 0.002
Control paper – Odour stimulus:	z = -3.20, p = 0.013	x Odour stimulus – Visual stimulus:	z = 5.47, p < 0.001
Control paper – x Visual stimulus:	z = -4.88, p < 0.001	Odour stimulus – x Visual stimulus:	z = -1.63, p = 1
Control paper – Visual stimulus:	z = -6.63, p < 0.001	Odour stimulus – Visual stimulus:	z = 3.38, p = 0.007
x Odour stimulus – Odour stimulus:	z = 2.02, p = 0.432	x Visual stimulus – Visual stimulus:	z = 1.77, p = 0.764

Figure S1: Representative volatile collection of *A. podagraria* umbells (Dynamic headspace collection 50 mg HayeSep 60-80 mesh, 1.5 hours)

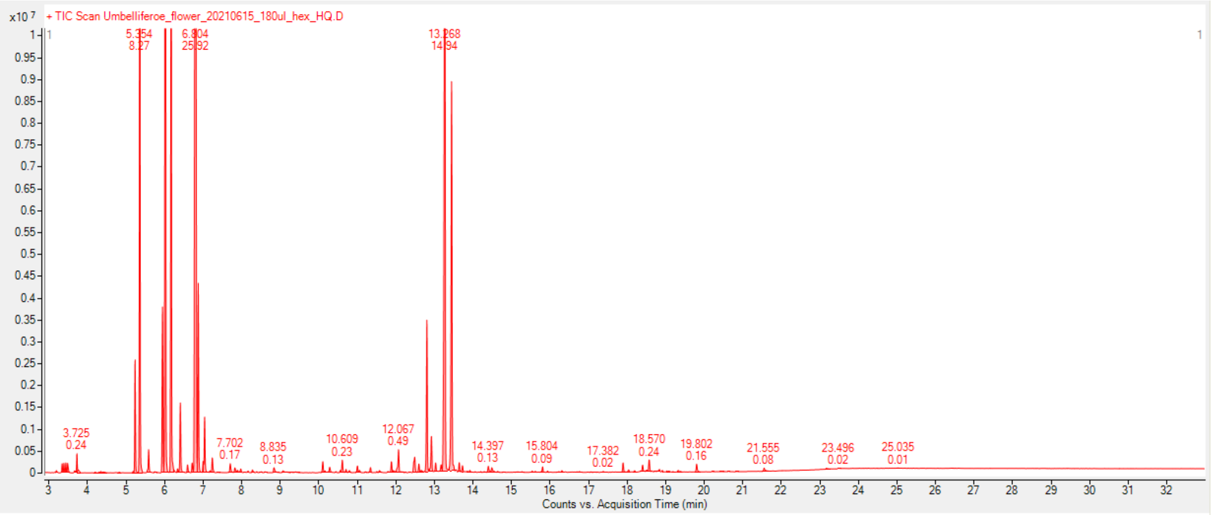

Figure S2: Representative volatile collection of dried A. podagraria umbellet, used as visual stimuli in experiments (DVB/PDMS/CAR coated fibers (StableFlex, 50/30 μm, Supelco, Sigma-Aldrich, Bellefonte, PA, USA, 1.5 hour volatile sampling) (take note that the scale is identical to Figure S1) (detected peaks are siloxane contamination from SPME fiber, and HP-5 column)

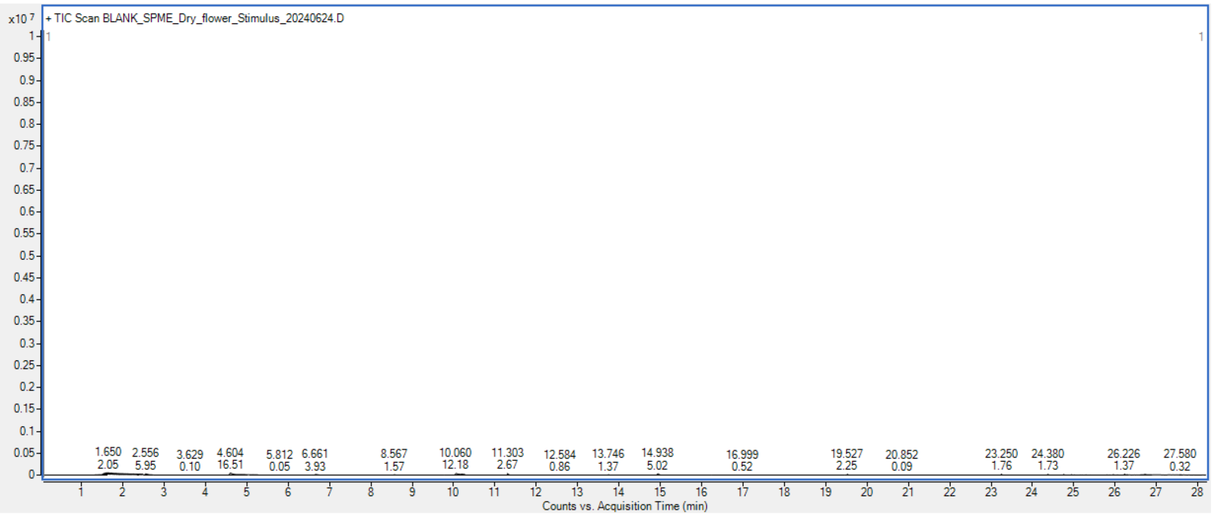

Supplement: Supplementary file 1 — Supplementary Information. [file 41598_2025_22240_MOESM1_ESM.pdf]
